# Sequential central retinal artery occlusions associated with cryoglobulinemia

**DOI:** 10.1186/s40942-022-00423-y

**Published:** 2023-03-22

**Authors:** Patrick Wang, Pushpinder Kanda, Yao Wang, Manpartap Bal

**Affiliations:** 1 Department of Ophthalmology, Queen’s University, Kingston, ON Canada; 2 Department of Ophthalmology, The University of Ottawa Eye Institute, Ottawa, ON Canada; 3 Division of Ophthalmology, Eastern Health, Eye Clinic, Health Sciences Centre, 300 Prince Philip Dr, St. John’s, NL A1B 3V6 Canada

**Keywords:** Cryoglobulinemia, Mixed cryoglobulinemia, Central retinal artery occlusion

## Abstract

**Background:**

Cryoglobulinemia, the presence of serum cryoglobulins which are immunoglobulins or complement components that precipitate at temperatures below 37 °C, commonly present with cutaneous manifestations initially, but are more rarely associated with ocular manifestations. To our knowledge, we report the first case of a patient presenting with sequential central retinal artery occlusion (CRAO) associated with cryoglobulinemia.

**Case presentation:**

A 69-year-old female with a history of indolent B-cell lymphoma associated cryoglobulinemia, treated hepatitis B infection and CRAO in the left eye presented with acute vision loss and diffuse retinal whitening with a cherry red spot in her right eye, suggestive of sequential CRAO. Laboratory studies revealed a cryocrit of 55% (normal  < 1%), elevated titres of cryoglobulin IgG at 1.98 g/L and cryoglobulin IgM at 3.78 g/L (normal  < 0.3 g/L)^9^, and elevated kappa free light chain at 283.5 mg/L (normal  < 0.06 g/L). Such elevated tires of cryoglobulins in the context of the patient’s CRAO raised suspicion of cryoglobulinemia associated CRAO. The patient was promptly referred to rheumatology and oncology and was admitted for treatment including intravenous methylprednisone, rituximab and bendamustine chemotherapy.

**Conclusions:**

We report a patient with a complex medical history presenting with significant vision loss due to a sequential CRAO likely associated with cryoglobulinemia. Although a direct relationship between cryoglobulinemia and CRAO cannot be confirmed in this case, it highlights the importance of considering cryoglobulinemia in high-risk patients with prior history of hematological malignancy or chronic hepatitis infection.

## Introduction

Cryoglobulins are serum immunoglobulins or complement components that precipitate at temperatures below 37 °C and solubilize upon rewarming. The presence of these cryoglobulins in serum, or cryoglobulinemia, results in the deposition of cryoglobulin-containing immune complexes in small to medium-sized blood vessels. Reports of ocular manifestation of cryoglobulinemia include, (1) retinal vein occlusion [1], (2) retinal artery occlusion [2], (3) Purtscher-like retinopathy [3], and (4) serous macular detachment [4]. While other vasculitic disorders such as systemic lupus erythematosus and polyarteritis nodosa are more commonly associated with central retinal artery occlusion (CRAO), the association with cryoglobulinemia is rare, making immediate diagnosis and clinical management challenging [2, 5–7]. To our knowledge, we report the first case of a complex patient presenting with sequential CRAO associated with cryoglobulinemia.

## Case presentation

**Fig. 1:**
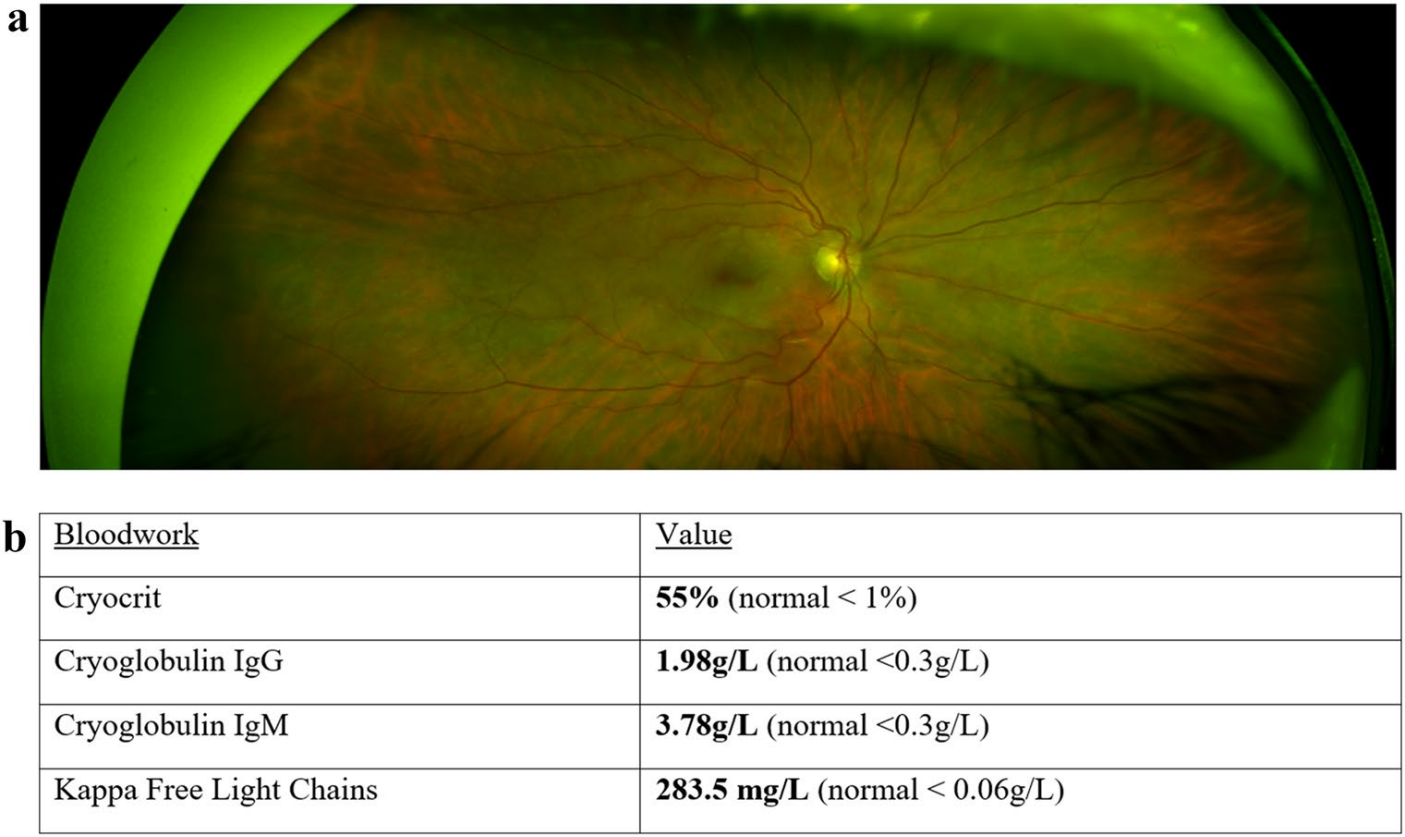
**A** Color fundus photograph of the right eye demonstrating retinal whitening in the posterior pole and a cherry-red spot suggestive of an acute central retinal artery occlusion. **B** Initial cryoglobulinemia work-up done at the time of presentation showcasing elevated titres of cryoglobulins

**Fig. 2:**
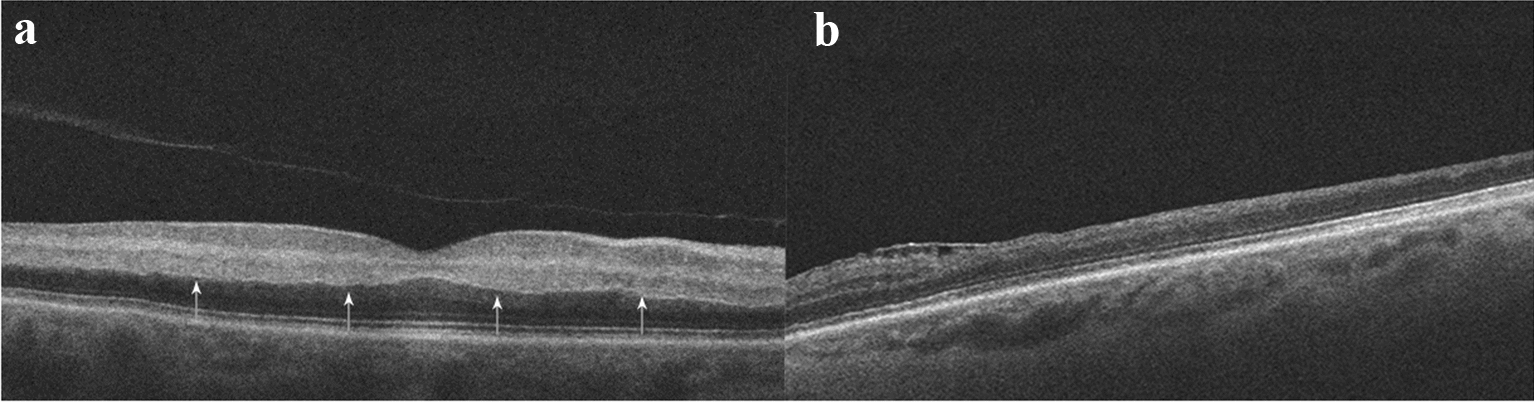
**A** SD-OCT of the right eye demonstrating increased hyper-reflectivity and thickening of the inner retina. A prominent middle limiting membrane sign is present (arrows) which can be seen in acute retinal ischemia. **B** SD-OCT of the left eye showing inner retinal thinning and atrophy indicative of a remote chronic central retinal artery occlusion

A 69-year-old female with a past ocular and medical history significant for remote CRAO in the left eye, indolent B-cell lymphoma with associated cryoglobulinemia, and treated hepatitis B infection presented with flashes and acute decreased central vision in the right eye over 1 day. Blood pressure measured in the clinic was 240/110. Ophthalmic examination of the right eye demonstrated a vision of counting fingers, normal intraocular pressure, and normal anterior segment. A grade 3 rapid afferent pupillary defect and vision of hand motions was observed in the left eye. Dilated fundus examination in the right eye revealed superficial retinal opacification of the posterior pole with a cherry red spot (Fig. 1). External examination of the patient’s extremities revealed rashes on her lower legs and purple discoloration of her feet. Spectral domain optical coherence tomography (SD-OCT) of the right eye revealed increased inner retinal hyperreflectivity and thickening with a prominent middle limiting membrane sign (Fig. 2A). Fluorescein angiography was deferred due to the patient’s impaired renal function. SD-OCT of the old CRAO in the left eye shows inner retinal thinning and atrophy (Fig. 2B).

Given the patient’s complicated medical history and elevated blood pressure at presentation, multiple aetiologies were considered including embolic, vascular, vasculitic, and neoplastic causes. She underwent a complete inflammatory and neoplastic workup including complete blood count, erythrocyte sedimentation rate, C-reactive protein, antinuclear antibody, rheumatoid factor, anti–β2 glycoprotein, cardiolipin antibody, hepatitis B serology, C3 complement, C4 complement, antineutrophil cytoplasmic antibody, angiotensin converting enzyme, serum free light chains, and serum electrophoresis. Additional testing included carotid angiogram, echocardiography, and temporal artery biopsy. Laboratory studies revealed a cryocrit of 55% (normal  < 1%), elevated titres of cryoglobulin IgG at 1.98 g/L and cryoglobulin IgM at 3.78 g/L (normal  < 0.3 g/L) [8], and elevated kappa free light chain at 283.5 mg/L (normal  < 0.06 g/L). The rest of her workup testing was unremarkable. Thus, detection of elevated tires of cryoglobulins raised suspicion of cryoglobulinemia associated CRAO.

**Fig. 3:**
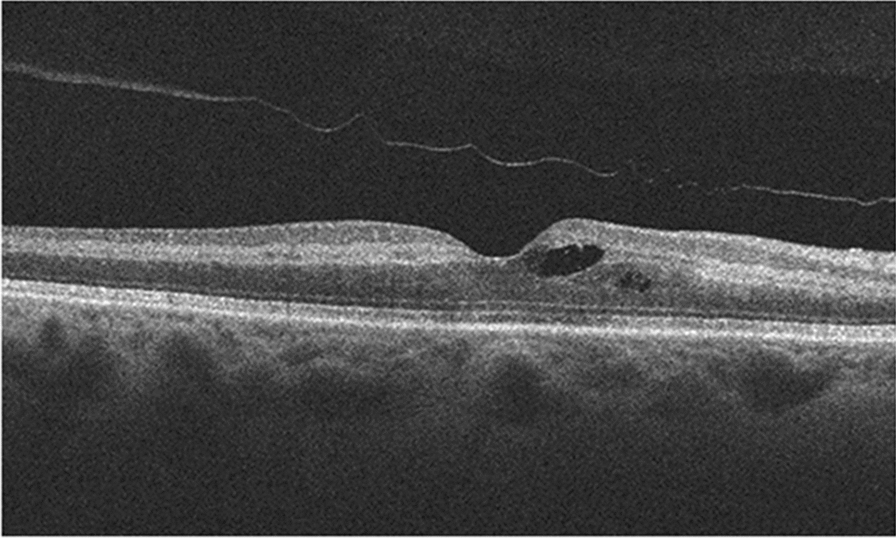
SD-OCT of the right eye 10 days following acute central retinal artery occlusion. Mild intraretinal cysts in the parafoveal region can be seen following some regression of inner retinal thickening

As such, the patient was promptly referred for a rheumatology consult, oncology follow-up, and admitted by internal medicine to receive intravenous methylprednisolone 1 g daily for 3 days. Rheumatology proposed that patient’s CRAO was likely associated with cryoglobulinemia vasculitis secondary to her kappa light chain restricted B-cell lymphoma. As such, she was started on rituximab as well as bendamustine chemotherapy for her lymphoma. Intravenous immunoglobulin and plasma exchange were also considered as potential treatment options but were refused by the patient due to religious beliefs as a Jehovah’s Witness. Unfortunately, visual acuity of the right eye was diminished to counting fingers at later follow up (day 10) with SD-OCT revealing resolving inner retinal thickening (Fig. 3).

## Discussion

Type I cryoglobulinemia, characterized by the presence of single monoclonal immunoglobulins (typically IgG or IgM) that precipitate with cold temperatures and dissolve with rewarming often occurs in the setting of monoclonal gammopathies or hematological malignancies such as multiple myeloma (11–20%) and Waldenström macroglobulinemia (18–33%) [9]. Lymphoproliferative diseases such as chronic lymphocytic leukemia and some lymphomas, especially marginal zone lymphoma and mantle zone lymphoma are also associated with 11–20% of cases of detectable type 1 cryoglobulins. Mixed cryoglobulinemia, including type II and type III cyroglobulinemia, are composed of monoclonal and/or polyclonal immungolublins of all isotypes with the most common forms combining monoclonal IgM and polyclonal IgG [9]. Chronic hepatitis C virus (HCV) infection is responsible for 50% of mixed cryoglobulinemias and 35% of HCV-infected patients have a cryoglobulinemia [10]. More rarely, mixed cryoglobulins are detectable in cases of B-cell lymphoma and after other infections such as hepatitis B or human immunodeficiency virus, as well as other bacterial and parasitic infections. The extrahepatic clinical manifestation of chronic hepatic infection includes the development of B cell lymphoma secondary to chronic activation of B cells [11].

Given the microheterogeneity and elevation of both monoclonal IgM and IgG components, the patient we present most likely suffered from mixed cryoglobulinemia in the context of her B-cell lymphoma and chronic hepatitis B infection. Patients with mixed cryoglobulinemia commonly present with cutaneous manifestations initially, such as palpable purpura, arthralgia, and weakness (Meltzer’s triad), and more rarely, ophthalmic involvement [1, 2]. It is important to educate patients that cold exposure as well as protracted standing, exercise, depilation, drugs, and infections are known triggers for flares of the disease [9].

Cryoglobulins can precipitate in vessels, increase serum viscosity, and mediate immune-complex vasculitis producing a wide array of symptoms including digit ischemia, skin necrosis, Raynaud’s phenomenon, glomerulonephritis, cerebral vascular event, and peripheral neuropathy [9]. While CRAO is a well-known complication of paraproteinemias and other hyperviscosity states, it is rare in the setting of cryoglobulinemia, and unreported for sequential bilateral CRAO. Given the patient’s age and risk factors, multiple causes were considered including embolic and other vascular etiology (e.g. giant cell arteritis). However, given her long standing history of cryoglobulinemia and previous suspected cryoglobulinemia-induced CRAO, the possibility of vessel occlusion secondary to immunoglobulin precipitation could not be excluded. We believe that the mechanism of CRAO may have resulted from the elevated circulating levels of monoclonal cryoglobulin IgG and IgM which formed immune complexes that deposited into pre-capillary arterioles causing embolic occlusion resulting in CRAO. The patient’s presentation of hypertensive urgency likely played a synergistic role in developing CRAO as hypertension has been reported as a significant risk factors in up to 33% of cases leading to CRAO [12]. In addition, there was no clinical evidence to strongly suggest other causes of CRAO such as giant cell arteritis, carotid stenosis or a cardioembolic source.

In summary, we report a patient with a complex medical history presenting with significant vision loss due to a sequential CRAO likely associated with cryoglobulinemia. Although a direct relationship between cryoglobulinemia and CRAO cannot be confirmed in this case, it highlights the importance of considering cryoglobulinemia associated CRAO, especially in high-risk patients with prior history of hematological malignancy or chronic hepatitis infection. As such, it is important for the ophthalmologist to collaborate with other specialties (i.e. internal medicine, rheumatology, etc.) for prompt initiation of treatment when suspecting cryoglobulinemic vasculitis.

## Data Availability

Due to the nature of this research, participants of this report did not agree for their data to be shared publicly, so supporting data is not available.

## Abbreviations

CRAO: Central retinal artery occlusion
SD-OCT: Spectral domain optical coherence tomography
HCV: Hepatitis C virus
